# Self-amplifying mRNA seasonal influenza vaccines elicit mouse neutralizing antibody and cell-mediated immunity and protect ferrets

**DOI:** 10.1038/s41541-023-00747-2

**Published:** 2023-10-04

**Authors:** Michael Cheung, Cheng Chang, Raveen Rathnasinghe, Evan Rossignol, Yunfei Zhang, Annette Ferrari, Harsh Patel, Yanjun Huang, Michelle Sanchez Guillen, Tina Scalzo, Changkeun Lee, Gillis R. Otten, Ethan C. Settembre, Nedzad Music, Giuseppe Palladino, Yingxia Wen

**Affiliations:** CSL Seqirus, 225 Wyman Street, Waltham, MA 02451 USA

**Keywords:** Influenza virus, Preclinical research

## Abstract

Currently licensed influenza vaccines focus immune responses on viral hemagglutinin (HA), while the other major surface glycoprotein neuraminidase (NA) is not tightly controlled in inactivated vaccine formulations despite evidence that anti-NA antibodies reduce clinical disease. We utilized a bicistronic self-amplifying mRNA (sa-mRNA) platform encoding both HA and NA from four seasonal influenza strains, creating a quadrivalent influenza vaccine. sa-mRNA vaccines encoding an NA component induced the production of NA-inhibiting antibodies and CD4^+^ T-cell responses in both monovalent and quadrivalent formulations. Including NA in the vaccine enabled cross-neutralization against antigenically drifted strains and provided greater protection than HA alone upon A(H3N2) challenge in ferrets. These results demonstrate that next-generation bicistronic sa-mRNA vaccines expressing HA and NA induce potent antibodies against both viral coat proteins, as well as vaccine-specific cell-mediated immunity. When formulated as a quadrivalent seasonal influenza vaccine, the sa-mRNA platform provides an opportunity to increase the breadth of protection through cross-neutralizing anti-NA antibodies.

## Introduction

Seasonal influenza vaccination is an effective means of generating widespread immunity to limit the considerable burden of annual influenza epidemics. An effective anti-influenza immune response relies on two important components: the production of virus-neutralizing antibodies to prevent the attachment and release of infective virions and the generation of adaptive immune effector and memory cells, including CD4^+^ and CD8^+^ T cells and memory B cells. Vaccine efficacy has traditionally been assessed through the generation of inhibitory antibody responses against the viral coat protein hemagglutinin (HA), and the current generation of licensed quadrivalent influenza vaccines focuses immunity on the HA associated with four circulating seasonal strains, including two A and two B strains^1^. Humoral responses generated against vaccine strains are generally strong; however, mismatches due to antigenic drift from point mutations in the viral genome can substantially reduce vaccine efficacy against circulating strains^2,3^. This antigenic drift also limits the protection afforded by preexisting antibodies^4^.

The other major viral coat glycoprotein, neuraminidase (NA), is either entirely absent from or not quantified in vaccine formulations despite extensive evidence from both animal and human studies that NA-specific antibodies reduce influenza virus replication, shedding, and transmission and thus the occurrence of clinical disease^5,6^. Because changes in the HA and NA antigenic sites occur asynchronously^7,8^, structurally similar NAs may circulate for several seasons even as selective pressure drives HA antigens to rapidly mutate away from each other. Robust immunity to NA could compensate for reduced vaccine efficacy due to HA drift in circulating strains. Challenge studies in animal models have demonstrated that although antibodies to NA do not achieve sterilizing immunity, a higher NA inhibition (NAI) titer is associated with reduced viral load^9^. In humans, clinical studies have demonstrated that higher titers of NA-inhibiting antibodies in sera before exposure correlate with reduced disease duration and severity and protection from drifted strains^10,11^. Another benefit of targeting NA with seasonal influenza vaccines is cross-protection against potential pandemic viruses, as demonstrated against avian H5N1 influenza in mice immunized with N1 from a circulating human A(H1N1) strain^12^. An analysis of human sera revealed that some individuals bear antibodies capable of inhibiting avian strains of N1^12^, further suggesting that NA-specific antibodies could limit negative outcomes in the case of emergent pandemic influenza.

Another limitation of contemporary seasonal influenza vaccine consisting of HA subunits or recombinant HA is a failure to induce or boost robust CD8^+^ cytotoxic T-cell responses^13–15^. Because stimulation of CD8^+^ T cells occurs via antigen presented on MHC class I molecules, induction of strong CD8 vaccine responses depends on endogenously generated antigen, such as through vaccines based on live-attenuated viruses or nucleic acids. CD8^+^ T cells are known to play a critical role in antiviral immunity. Following infection, virus-specific CD8^+^ T cells become activated and begin the processes of expansion and differentiation to effector T cells, which produce antiviral cytokines, including interferon-γ (IFN-γ) and tumor necrosis factor-α (TNF-α), and mediate the killing of virus-bearing cells via granzyme and perforin release^16^. Following the resolution of the immune response, a subset of CD8^+^ T cells is retained as long-lived memory cells capable of rapid expansion upon secondary exposure to the virus^16^. In addition, CD8^+^ T cells may impart immunity against heterosubtypic strains^14^, underscoring the benefits of targeting cytotoxic T cells in seasonal influenza vaccines.

The advent of mRNA vaccines and their recent successful use during the SARS-CoV-2 pandemic have demonstrated the potential of mRNA technology to generate protective antiviral responses through both neutralizing antibodies and cell-mediated immunity^17,18^. Because the target protein is expressed endogenously, potential egg- and cell culture–derived antigenic adaptations associated with recombinant protein and subunit vaccines are avoided^19,20^. Self-amplifying mRNA (sa-mRNA) vaccines, which exhibit the benefits of mRNA vaccines, offer an opportunity to generate immunity to multiple viral proteins with a much-reduced RNA dose^21,22^, decreasing the potential for adverse events following immunization and providing a promising avenue for rapid and cost-effective vaccine production for both seasonal and pandemic preparedness^23,24^. In addition to encoding the target gene of interest, sa-mRNA constructs also contain replicase genes encoding an RNA-dependent RNA polymerase (RdRp) that amplifies the transcription of the construct and the antigen targets, leading to greater antigen expression per mRNA dose and extending the duration of expression^25^. Our next-generation sa-mRNA construct advances this technology by providing balanced expression of multiple genes of interest per sa-mRNA molecule, reducing the amount of mRNA and packaging lipid nanoparticles (LNP) needed for multiantigen vaccines^26^. In this study, we designed multiple sa-mRNA constructs expressing HA and/or NA from four clinically relevant influenza strains. We demonstrate that each bicistronic sa-mRNA LNP induced the production of specific, robust, and dose-dependent neutralizing antibodies to both HA and NA and reveal their capacity to generate humoral and cell-mediated immune responses when formulated together as a novel quadrivalent vaccine against seasonal influenza.

## Results

### Bicistronic sa-mRNA constructs induce the expression of HA and NA in vitro

**Fig. 1:**
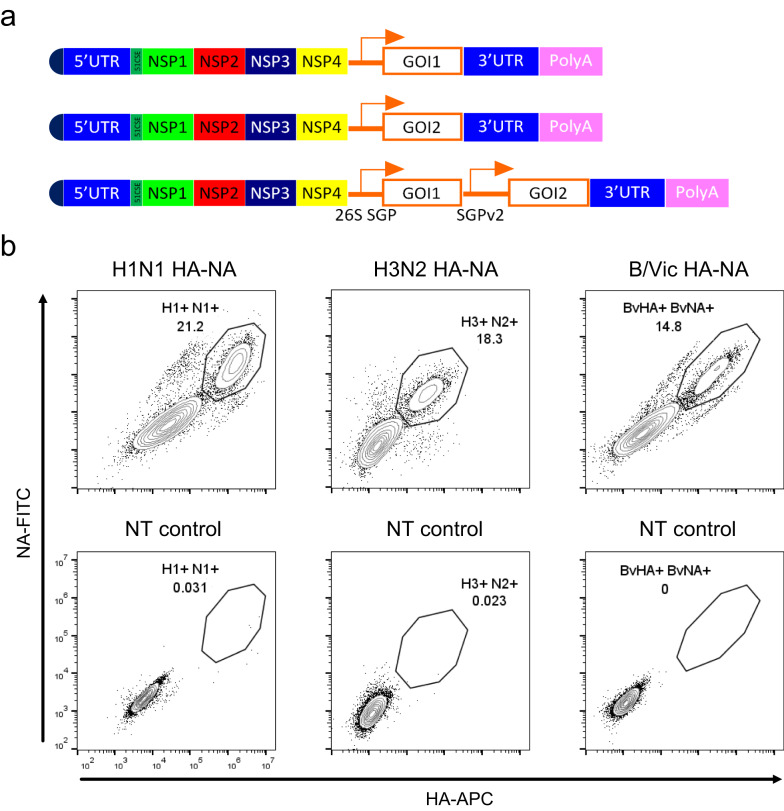
Monocistronic and bicistronic sa-mRNA constructs. **a** Monocistronic and bicistronic design schematic. UTR untranslated region, NSP, nonstructural protein, SGP subgenomic promoter, GOI gene of interest. **b** Expression of HA and NA following transfection with bicistronic sa-mRNA constructs, or in non-transfected (NT) control cells. Numbers indicate the frequency (%) of cells that fall within the gate.

To examine whether sa-mRNA could be used as a platform for a complex vaccine with multiple antigenic targets, such as a quadrivalent seasonal influenza formulation, we utilized our previously reported bicistronic sa-mRNA construct^25,26^, consisting of a series of four nonstructural proteins (NSPs) required to amplify the mRNA construct and two subgenomic promoter (SGP) sites, one for each gene of interest (GOI) (Fig. 1a). For these experiments, we developed four bicistronic constructs, including one for each seasonal influenza strain: A/Delaware/55/2019 (H1N1), A/Delaware/39/2019 (H3N2), B/Darwin/7/2019 (B/Vic), and B/Singapore/INFTT-16-0610/2016 (B/Yam). In each construct, HA was incorporated into the GOI1 site, while NA was inserted into the GOI2 site. In addition, we designed eight monocistronic sa-mRNA constructs to encode each HA or NA antigen alone for comparison (Fig. 1a). The expression of both antigens was evaluated in BHK-V cells by flow cytometry. As expected, transfection with bivalent sa-mRNA constructs produced HA and NA double-positive populations as the dominant positive population (Fig. 1b), except for the B/Yamagata sa-mRNA-HA-NA construct, which was not analyzed by flow cytometry due to the lack of a B/Yamagata NA-specific antibody.

### Quadrivalent sa-mRNA-HA-NA vaccine induced neutralizing antibody responses against four seasonal HA and NA in mice

**Fig. 2:**
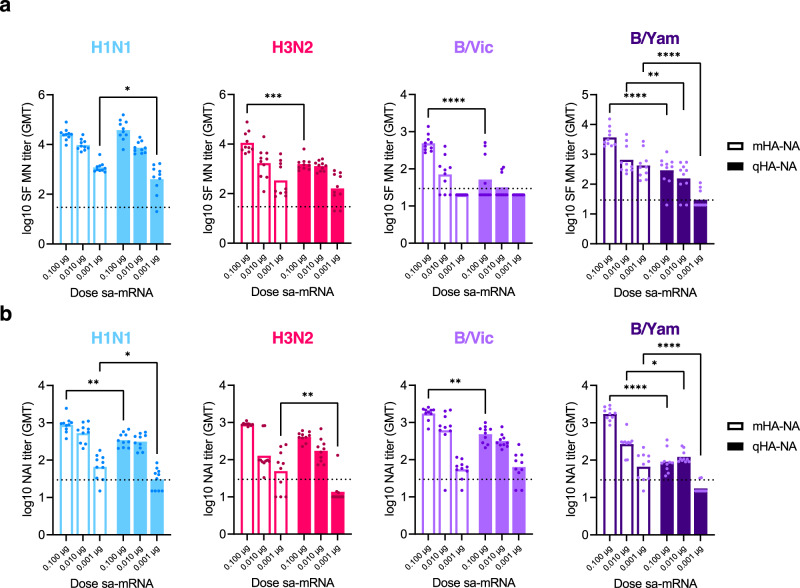
Quadrivalent bicistronic sa-mRNA vaccines potently induce the production of HA- and NA-neutralizing antibody titers. BALB/c mice (10 per group) were vaccinated twice, 3 weeks apart with the indicated dose and type of vaccine. HA-specific (**a**) and NA-specific (**b**) responses are compared across monovalent bicistronic (mHA-NA) and quadrivalent bicistronic mRNA vaccines. **a** HA-specific neutralizing antibody responses induced by monovalent bicistronic sa-mRNA vaccines in vivo were measured by a short-form microneutralization (MN) assay. **b** The NA-specific neutralizing antibody response induced in vivo was measured by enzyme-linked lectin assay (ELLA). mHA-NA and qHA-NA compared by two-way ANOVA, *p < 0.05, **p < 0.01, ***p < 0.001, ****p < 0.0001.

While we have reported on the robust immunogenicity of monovalent bicistronic sa-mRNA-HA-NA vaccines previously^27^, it remains an open question whether the platform is capable of inducing immune responses to the eight antigenic targets necessary for a quadrivalent seasonal influenza vaccine encoding both HA and NA. To address this, we immunized mice with a quadrivalent bicistronic sa-mRNA HA-NA vaccine against all four seasonal influenza strains testing three doses of RNA, including 0.1 µg, 0.01 µg, or 0.001 µg per construct. In addition, some mice received only one of the four monovalent sa-mRNA-HA-NA vaccines (mHA-NA) as a control. Three weeks after the second dose we compared monovalent and quadrivalent vaccines for neutralizing HA- and NA-specific antibody responses by enzyme-linked immunosorbent assay (ELISA), short-form microneutralization (SF MN), and NAI. The production of vaccine-specific memory B cells was assessed by enzyme-linked immunosorbent spot (ELISpot). Quadrivalent HA-NA (qHA-NA) vaccines expressing eight antigen targets produced functional antibody titers above the threshold of detection against A(H1N1), A(H3N2), and B/Yamagata across three doses, as indicated by SF MN assays, which are primarily used to measure anti-HA antibody neutralizing capability (Fig. 2a), and NAI (Fig. 2b). HA-specific neutralizing antibodies against B/Victoria were reduced in the qHA-NA vaccine group compared to the mHA-NA group (Fig. 2a), although B/Victoria NAI titers were similar (Fig. 2b). The qHA-NA vaccine stimulated anti-HA and anti-NA immunoglobulin G (IgG) responses which were similar to each corresponding mHA-NA group (Supplementary Fig. 1a and b). Moreover, immunization with the qHA-NA vaccine (0.1 µg dose) established antigen-specific splenic memory B cells against all four vaccine strains (Supplementary Fig. 2). The inclusion of six additional targets in the quadrivalent formulation resulted in a modest reduction in the neutralizing antibody response against A(H1N1) and A(H3N2) HA and NA. The difference between the quadrivalent and monovalent vaccines was more prominent against B/Victoria, which showed significantly reduced SF MN titers at all three doses (Fig. 2a), and against B/Yamagata NA, which similarly exhibited reduced titers in the quadrivalent vaccine groups. The impact of this effect on protective immunity is unknown, as the quadrivalent vaccine-induced NAI titers were above the threshold of detection against all four strains of influenza when both the 0.1 and 0.01 µg doses were used. Reduced neutralizing titers resulting from additional antigenic targets are expected and have been observed with other vaccine platforms, such as adjuvanted subunit vaccines, in comparisons of monovalent and multivalent formulations (Supplementary Fig. 3). These results will help establish the best dose to use for the development of future quadrivalent vaccines and suggest there may be a need for slightly higher doses to compensate for reduced response to influenza B strains.

### Immunization with quadrivalent sa-mRNA vaccines induces the production of influenza-specific CD4 and CD8 T cells

**Fig. 3:**
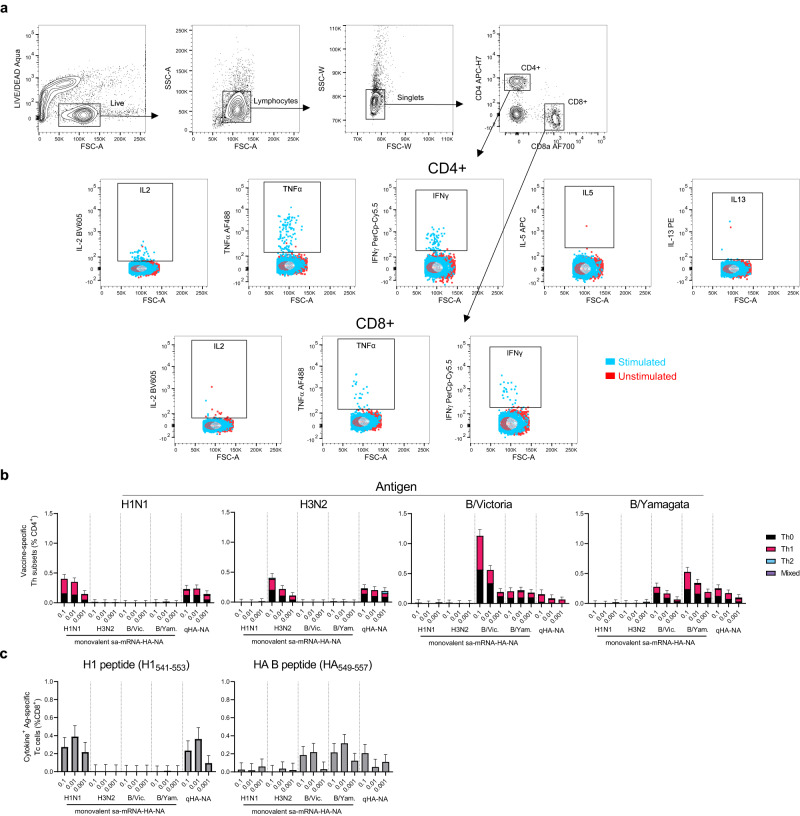
Bicistronic sa-mRNA vaccines induce the production of virus-specific helper and cytotoxic T cells. **a** Gating strategy for antigen-specific CD4^+^ and CD8^+^ splenocytes. **b** Splenic CD4^+^ T cells from immunized mice were pooled (5 per group) and stimulated with vaccine monobulks ex vivo. T cells producing the cytokines IL-2, TNF-α, INF-γ, IL-5, and IL-13 were identified by flow cytometry. **c** CD8^+^ T cells from immunized mice were stimulated with peptides from H1N1, B/Victoria (B/Vic), or B/Yamagata (B/Yam), and cells producing IL-2, TNF-α, and INF-γ were quantified by flow cytometry. Bars represent the mean cytokine-positive CD4 subsets (**b**) or CD8 T cells (**c**) and T-bars the 95% confidence interval, which is calculated from duplicate measurements on pooled spleens and indicates the precision of the measurement.

To analyze the T-cell responses in vaccinated mice, we assessed T-cell responses in splenocytes 21 days after the second vaccination. Splenocytes were stimulated ex vivo by vaccine-homologous subunit antigens, which were predominantly HA with an unquantified amount of NA, then characterized after 6 h of stimulation by intracellular cytokine staining and multiparameter flow cytometry (Fig. 3a). In response to homologous subunits, the CD4^+^ T cells that were produced through the vaccine (Fig. 3b) produced IFN-γ, TNF-α, and interleukin-2 (IL-2) with little or no IL-5 or IL-13 expression, indicating a predominantly Th1 response. The proportions of CD4^+^ cells produced through the vaccine were similar between groups administered the different doses and between those administered monovalent and quadrivalent vaccines, with the exception of B/Victoria-specific CD4^+^ cells, which were reduced in the qHA-NA compared to the monovalent B/Victoria vaccine group. Similarly, influenza A H1 and influenza B HA-specific CD8^+^ T cells were detected after both quadrivalent and monovalent administration of the bicistronic vaccines at similar levels (Fig. 3c). The levels of influenza A H3- and NA-specific CD8^+^ cells could not be measured in our study due to the lack of appropriate CD8^+^ T cell antigen. The CD8^+^ responses to antigen predominantly involved the expression of IFN-γ and/or TNF-α, with low levels of IL-2. Neither CD4^+^ nor CD8^+^ T cells showed cross-reactivity among A(H3N2) and A(H1N1) subtypes, which were 41.9% and 41.6% identical at the amino acid level for HA and NA, respectively. Cross-reactivity was observed between B/Yamagata and B/Victoria, which are 92.5% and 92.9% identical for HA and NA, respectively. In summary, these results indicate that quadrivalent vaccination is capable of inducing CD4^+^ and CD8^+^ T-cell responses against these antigens.

### Bicistronic quadrivalent vaccination induced immune responses that were similar to the responses to monocistronic quadrivalent or octavalent vaccines

**Fig. 4:**
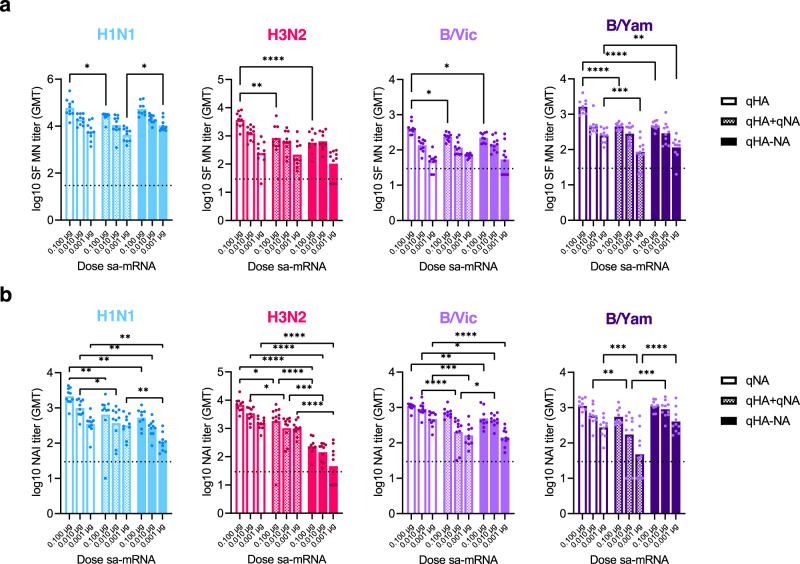
Monocistronic HA or NA and bicistronic HA-NA sa-mRNA vaccines stimulate the production of neutralizing antibodies. **a** The levels of neutralizing HA-specific antibodies induced by monocistronic or bicistronic sa-mRNA vaccines were measured by MN of vaccine strains in the Focus forming assay (FFA) MN assay. **b** Inhibition of NA by neutralizing antibodies was assayed by ELLA. B/Vic, B/Victoria; B/Yam, B/Yamagata. 2-way ANOVA, *p < 0.05, **p < 0.01, ***p < 0.001, ****p < 0.0001.

In inactivated or recombinant seasonal influenza vaccines, HA has been used as either the primary or only viral component of seasonal influenza subunit vaccines. Therefore, we immunized mice with quadrivalent monocistronic or bicistronic sa-mRNA vaccines to determine the benefits and tradeoffs of a qHA-NA approach compared with quadrivalent HA-only (qHA) and NA-only (qNA) vaccines. In addition, we tested an octavalent formulation, consisting of four monocistronic sa-mRNA constructs expressing seasonal HAs and four monocistronic sa-mRNA constructs expressing seasonal NAs (qHA+qNA). With a few exceptions, the neutralizing anti-HA antibody response, measured by SF MN, was similar between groups receiving qHA regardless of whether qNA was included in a separate or the same sa-mRNA molecule (Fig. 4a). Even at the low dose of 0.001 µg mRNA, the geometric mean of the neutralization titer was above the threshold of detection for all four seasonal strains. NAI was similar among the three vaccine groups for influenza A N1 and both B strain NAs (Fig. 4b), with the response to influenza A N2 being reduced in the qHA-NA groups. Taken together, these results show that with the addition of NA, either in an additional cistron or on a second monocistronic construct, neutralization of both HA and NA can be achieved with limited loss of titer.

### The quadrivalent seasonal bicistronic sa-mRNA influenza vaccine provides expanded cross-neutralization through an NA-specific antibody response in vitro

**Fig. 5:**
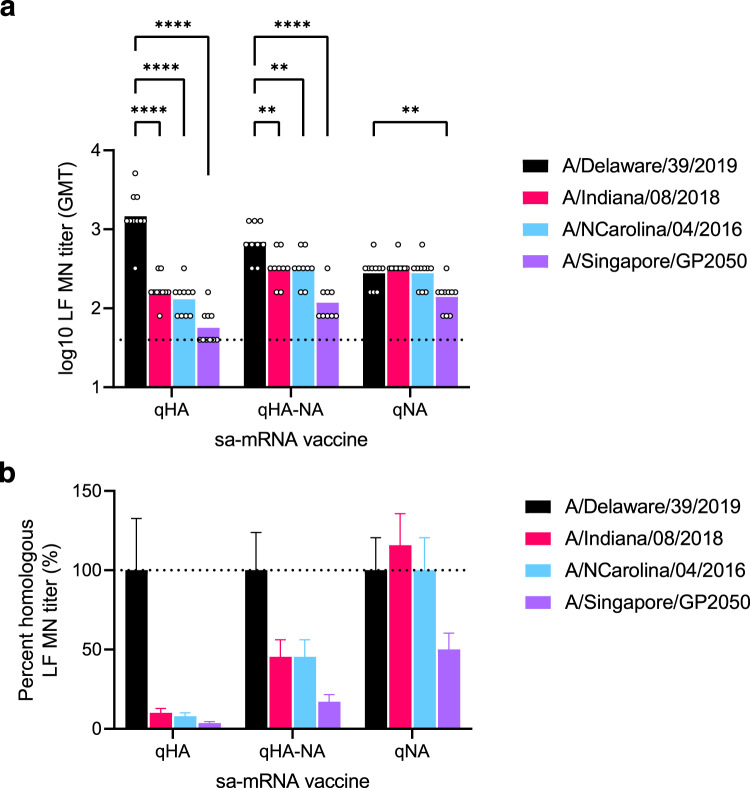
Quadrivalent bicistronic sa-mRNA vaccines induce the production of cross-neutralizing NA-specific antibodies. **a** The NA-specific neutralizing antibody response was measured by a long-form (LF) MN assay. **b** Cross-neutralizing response against heterologous A(H3N2) viruses are expressed as percent homologous (H3N2 A/Delaware/39/2019) LF MN titer (mean ± SEM). Titers against heterologous strains compared to A/Delaware/39/2019 by two-way ANOVA, **p < 0.01, ***p < 0.001, ****p < 0.0001.

One anticipated benefit of targeting NA in a seasonal influenza vaccine is the potential for extending protection to target heterologous strains. To examine potential cross-neutralization of heterologous strains due to anti-NA antibodies, we utilized a long-form microneutralization assay. As heterologous strains, we selected historical seasonal A(H3N2) viruses with varying phylogenic distances (Supplementary Fig 4): A/Indiana/08/2018, A/North Carolina/04/2016, and A/Singapore/GP2050/2015 (Fig. 5a). We found that immunization with a 0.1 µg dose of the qHA vaccine containing H3 from A/Delaware/39/2019 failed to effectively neutralize all three HA antigen-drifted heterologous strains ( ≥ 90% reduction in titer, Fig. 5b). In contrast, immunization with qNA containing N2 from A/Delaware/39/2019 resulted in LF MN titers against both A/Indiana/08/2018 and A/North Carolina/04/2016 that were equivalent to those of the vaccine strain, while the LF MN titer was 50% lower against the A/Singapore/GP2050/2015 strain (Fig. 5b), which showed more distant N2 drift (Supplementary Fig. 4b). Similarly, when we incorporated NA in our quadrivalent bicistronic design, the mice immunized with 0.1 µg of the qHA-NA vaccine had a much lower reduction (55%) in the LF MN titer against A/Indiana/08/2018 and A/North Carolina/04/2016 and an 83% reduction in titer against A/Singapore/GP2050/2015 H3N2 virus (Fig. 5b), with a geometric mean titer (GMT) similar to that in the qNA group (Fig. 5a). For all three heterologous A(H3N2) viruses, the qHA-NA and qNA groups exhibited higher cross-neutralizing titers than the qHA group (Fig. 5a), supporting the value of including NA in future quadrivalent influenza vaccines.

### Bicistronic sa-mRNA vaccines encoding HA and NA of seasonal A(H3N2) influenza viruses confer enhanced protection in a ferret challenge model

**Fig. 6:**
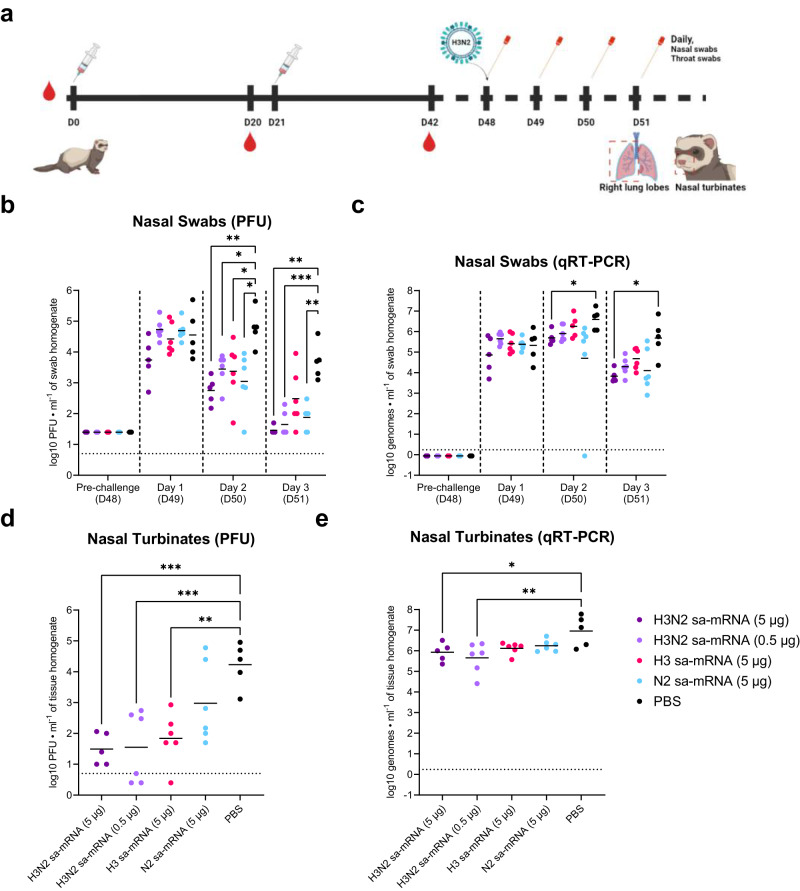
Bicistronic sa-mRNA vaccines targeting seasonal A(H3N2) protects ferrets from influenza challenge. **a** Experimental design for ferret protective study included a prime-boost vaccination regimen with two doses 3 weeks apart with pre- and post-immunization bleeds. Animals were challenged with 10^6^ TCID_50_ of A/Delaware/39/19 A(H3N2) per animal, and nasal swabs were taken daily during the challenge phase. Nasal turbinates and lung tissues were harvested 3 days post infection. Viral load in nasal swabs were determined by plaque assays (**b**) and qRT-PCR (**c**). Viral load in homogenized nasal turbinates was established by plaque assays (**d**) and qRT-PCR (**e**). Each dot represents one animal and data are presented as geometric means. Log-transformed nasal swab data were analyzed by using a two-way analysis of variance (ANOVA) with multiple comparisons using Dunnett’s adjustment. Log-transformed nasal turbinate data was analyzed by using a one-way ANOVA with multiple comparisons using Dunnett’s multiple comparisons. *p < 0.05, **p < 0.01, ***p < 0.001.

The effectiveness of bicistronic sa-mRNA vaccine effectiveness was next tested in the well-established ferret influenza challenge model^28^, commonly used to evaluate the protective capacity of a vaccination regimen and potential for severe outcomes for hospitalized patients^29^. As our previous results with H3N2 sa-mRNA vaccines indicated similar immunogenicity of monovalent and quadrivalent formulations (Fig. 2, Supplementary Fig. 1) and to focus on the effect of including both HA and NA while avoiding cross-subtype responses, monovalent vaccination was chosen over quadrivalent formulation for this study. Ferrets were primed and boosted with sa-mRNA vaccines encoding H3, N2, or both in a bicistronic construct (Fig. 6a). Four weeks after the second immunization ferrets were challenged with A(H3N2) virus. Daily infectious virus titers in the upper respiratory tract were quantified by plaque assays from nasal swabs. Peak viral loads for vaccinated animals occurred at day 1 post infection (D49), which subsided by each consecutive day, whereas peak viral load in mock immunized animals occurred at day 2 post challenge (D50) (Fig. 6b). By 2 days post challenge (D50), all vaccinated animals, regardless of the vaccine construct, had significantly lower viral titers compared to mock immunized animals, between 1 and 2 logs below the control (Fig. 6b). By day 3 post infection (D51), most immunized animals had virus titers near or below the limit of detection, while viral loads in mock immunized animals were in the range of 10^3^ PFU/mL (Fig. 6b). Ferrets that received 5 µg of bicistronic H3N2 sa-mRNA vaccines had a plaque-forming unit (PFU) titers that trended lower compared to all groups at each time point post challenge, although these differences were not statistically significant (Fig. 6b). Similarly, quantitative reverse transcription polymerase chain reaction (qRT-PCR), as a more sensitive approach, from nasal swabs indicated lower virus genome copies in all immunized groups on day 2 post challenge (D50), when viral genomes peaked in mock immunized animals (Fig. 6c). Recovery of live virus from nasal turbinates demonstrated significantly less infectious virus 3 days post challenge in all vaccinated groups except for animals that received the monocistronic sa-mRNA vaccine expressing N2 alone, although 4 of 6 ferrets in that group had more than a tenfold reduction in virus titer versus mock immunized animals (Fig. 6d). In addition, measurement of viral genome copies from nasal turbinates indicated that all vaccinated groups had lower total viral load compared to mock immunized animals (Fig. 6e). Ferrets that received bicistronic H3N2 sa-mRNA vaccines had the lowest nasal turbinate viral load compared to all groups, even at the low (0.5 µg RNA) dose (Fig. 6d,e). These data clearly indicate the benefit of including NA in the sa-mRNA vaccine in that it provides an enhanced protective immunity in the upper respiratory tract compared to HA alone. Beyond the upper respiratory tract, viral loads from throat swabs indicated similar results for vaccinated groups, particularly at 1 day post challenge, wherein both infectious and total viral loads were significantly lower in vaccinated animals compared to animals that received a mock vaccine (Supplementary Fig. 5a,b). Viral titers in lung tissues suggested no detectable infectious virus by day 3 post challenge (D51) for all animals except for 2 animals from the mock immunized group (Supplementary Fig. 5c). However, qRT-PCR showed vaccinated groups had lower viral genome in the lung versus mock immunized animals and several vaccine recipients had no detectable amounts of viral genomes from lung tissue (Supplementary Fig. 5d). In addition, sera from ferrets immunized with N2-containing sa-mRNA vaccines had cross-neutralizing antibodies against heterologous A(H3N2) strains, measured by LF MN (Supplementary Fig. 6), resembling our previous results in mice (Fig. 5). Taken together, this clinically relevant ferret-challenging model indicated an enhanced infectious and total virus clearance by the addition of NA using the bicistronic sa-mRNA approach.

## Discussion

Current licensed inactivated influenza vaccines focus on eliciting immunity against the HA protein, resulting in reduced vaccine efficacy when the HA associated with the vaccine and circulating strains differ due to antigenic drift or mismatch^30,31^. In contrast, the second major viral surface protein, NA, evolves more slowly^7,8^, and vaccines targeting NA may have increased breadth of protection^32–35^. Here, we demonstrate that sa-mRNA vaccines containing an NA component potently induce the production of NA-inhibiting antibodies in both monovalent and quadrivalent formulations. Furthermore, we demonstrate the in vivo enhanced efficacy of a bicistronic sa-mRNA vaccine expressing both HA and NA in a ferret challenge model. Immunization with sa-mRNA vaccines resulted in reduced viral loads in the upper respiratory tract, which is the primary site of A(H3N2) replication in this model^36,37^. In addition, we found reduced replication-competent virus in the throats and lungs of vaccinated ferrets, although internal studies and reports in the literature have demonstrated tissue tropism of most seasonal A(H3N2) viruses is limited to the ferret upper respiratory tract^37,38^. Although not statistically significant, animals that received the bicistronic sa-mRNA vaccine with both an HA and NA component bore consistently lower viral loads measured by PFU and qRT-PCR in both their upper and lower airways versus HA or NA alone, even 1 day post challenge or when the sa-mRNA dose was 10-fold lower, suggesting the additive protection afforded when both viral components were included.

In addition to neutralizing the vaccine strain, we demonstrated that anti-N2 antibodies generated using mRNA from the H3N2 A/Delaware/39/2019 strain circulating during the Northern Hemisphere (NH) 2020-2021 season were equally effective against strains circulating in the previous NH2019-2020 and NH2018-2019 seasons (A/Indiana/08/2018 and A/North Carolina/04/2016, respectively) and still maintained detectable neutralizing capacity against a strain from NH2017-2018 (A/Singapore/GP2050/2015). This finding was in contrast to the dramatic reduction in the cross-neutralizing capacity of anti-H3 antibodies, likely due to the more rapid, season to season antigenic drift of the H3 protein compared with N2. Sera from H3N2 sa-mRNA immunized ferrets recapitulated these results, and we have reported similar anti-NA-mediated cross-neutralization following immunization with sa-mRNA encoding the N1 of the seasonal H1N1 A/Delaware/55/2019 (NH20/21) against H5N1 pandemic influenza A/turkey/Turkey/1/2015^27,39^. Others have demonstrated that immunization with NA provides broad heterologous cross-protection against infection with both influenza A and B strains, albeit using different vaccine platforms^35,40,41^. While investigating cross-protective immunity against heterologous infective challenge was beyond the scope of these experiments, NAI titers have been shown to correlate with protection against heterologous influenza^42^. Therefore, a quadrivalent bicistronic sa-mRNA-HA-NA vaccine could provide both strong immunity against influenza A and B virus strains as well as broad protection against co-circulating and future seasonal strains.

In addition to vaccine-specific Th1-biased CD4^+^ T cell activation, the sa-mRNA-based vaccines induced influenza-specific CD8^+^ T-cell responses, which are absent when traditional inactivated and recombinant protein vaccines are used^15^. Because HA and NA are produced endogenously in the cytoplasm, the antigens are readily available for MHC-I presentation to virus-specific cytotoxic T cells. The presence of CD8^+^ T cells targeting conserved viral epitopes has been shown to compensate for the lack of cross-neutralizing influenza-specific antibodies and may provide cross-protection against symptomatic infection^43^.

In some cases, we found that increasing the antigen number per immunization by using a bicistronic sa-mRNA construct or a quadrivalent formulation of multiple sa-mRNA constructs resulted in reduced antibody titers and antigen-specific T cells, compared to the monocistronic or monovalent vaccine formulations, respectively. However, in most cases the magnitude of the reduction in antibody titers was small and therefore the immune consequences are unclear. Moreover, due to the self-amplifying nature of the vaccines, the dose range of sa-mRNA tested was very low when compared with the current generation mRNA-based vaccines. Therefore, it may be possible to compensate for a reduced titer by optimizing the dose of sa-mRNA, increasing antigen quantity with a modest increase in mRNA and LNP content^44^. Although we demonstrated that eight individual monocistronic sa-mRNA vaccines could be successfully delivered and induce neutralizing immunity, the use of four bicistronic HA-NA constructs was also successful. The benefits of the bicistronic quadrivalent approach include both a decrease in total amount of mRNA used per dose, reduced production of replicase proteins, and a lower amount of LNP components per immunization. These features may lessen the risk of adverse events, as well as the amount of materials and therefore the production cost per dose. As demonstrated throughout the ongoing SARS-CoV-2 pandemic, these considerations are vital when designing flexible and feasible vaccine platforms for future use, particularly for pandemic preparedness.

One limitation of the animal models used in this study was that they did not account for how preexisting immunity to influenza, which is prevalent in human populations, could affect both humoral and cellular responses generated by sa-mRNA vaccines. So-called original antigenic sin, or immunodominance, has been well described for anti-HA responses^45,46^ and may skew or otherwise alter responses to NA as well. Also, while our ferret studies have demonstrated reduced viral shedding during the early stage of influenza infection, future studies are needed to address other important vaccine-mediated effect, such as reduced duration of viral shedding and morbidity. In addition, while the presence of cross-neutralization by anti-NA antibodies would suggest protection against infection by antigenically drifted influenza in vivo, this phenomenon was not tested as a part of this study and future work will determine whether the NA component extends protection to heterologous strains, as indicated by the LF MN assay.

In summary, this work demonstrates that bivalent vaccines using sa-mRNA technology induce robust neutralizing and protective anti-influenza adaptive immune responses in vivo against both HA and NA, and these bivalent constructs can be co-formulated in a quadrivalent vaccine against four seasonal influenza strains, generating immunity to eight viral antigens simultaneously.

## Methods

### Construct design and cloning

All bicistronic constructs were generated based on the Venezuelan equine encephalitis virus (VEEV) TC-83 strain that has a chimeric 3′untranslated region (UTR) from Sindbis virus (SINV). SGPv2 contained the exact same full SGP sequence as described by Blakney, et al. (2018)^47^. To create a quadrivalent vaccine, the influenza A H1 and N1 were obtained from the A/Delaware/55/2019 isolate, influenza A H3 and N2 were obtained from the A/Delaware/39/2019 isolate; B/Victoria HA and NA were obtained from the B/Darwin/7/2019 isolate, and B/Yamagata HA and NA were obtained from the B/Singapore/INFTT-16-0160/2016 isolate. All clones and constructs were generated seamlessly by Gibson assembly into a sa-mRNA DNA construct for in vitro (IVT) that has a T7 promoter with G as the first nucleotide and a poly A (A = 37) tail.

### DNA linearization and IVT

Linearized DNA templates were enzymatically transcribed into RNA with T7 RNA polymerase (New England Biolabs, Ipswich, Massachusetts, USA), digested with Turbo DNAse (Life Technologies, Carlsbad, California, USA) to remove template DNA, and capped using a Vaccinia capping system (New England BioLabs). RNA from the transcription/capping reaction was purified by LiCl precipitation and frozen at −80 °C.

### sa-mRNA and LNP formulation

RNA was formulated into LNPs in citrate buffer using a proprietary ionizable lipid, 1,2-distearoyl-sn-glycero-3-phosphocholine (DSPC; Avanti Polar Lipid), 1,2-dimyristoyl-sn-glycero-3-phosphoethanolamine-N-[methoxy(polyethylene glycol)-2000] (PEG-DMG 2000; NOF America Corporation, White Plains, NY, USA), and cholesterol (Sigma-Aldrich, St. Louis, Mo, USA), dissolved in ethanol through a NanoAssemblr mixing instrument (Precision Nanosystems, Vancouver, Canada). These nanoparticles were buffer-exchanged into a Tris buffer with NaCl and sucrose by TFF, sterile-filtered, and stored at −80 °C for use in in vitro potency and in vivo immunogenicity experiments^26,27^.

### Cell culture, sa-mRNA transfection, and staining for flow cytometry

To evaluate the transfection efficiency of each sa-mRNA construct, baby hamster kidney V (BHK-V) cells, a proprietary derivative of BHK21 (ATCC, Manassas, Virginia, USA) were used. Cells were grown in high glucose Dulbecco’s modified eagle medium (DMEM; Thermo Fisher, Waltham, Massachusetts, USA) with 10% fetal bovine serum (FBS; HyClone, Cytiva, Marlborough, Massachusetts, USA), 2 mM L-glutamine, and 1% penicillin/streptomycin (P/S). Following transfection, high glucose DMEM with 1% FBS, 2 mM L-glutamine, and 1% antibiotic was used as the BHK-V outgrowth medium. For flow cytometry staining, monoclonal antibodies against HA and NA were labeled using Zenon kits (Thermo Fisher).

For sa-mRNA LNP transfection, 100 µL of LNP in room-temperature optiMEM (Thermo Fisher) with the indicated RNA concentrations was prepared. The 1E6 BHK-V cells were washed once with plain optiMEM and resuspended in 250 µL room-temperature optiMEM before being added to the LNP solution with gentle pipetting. The LNP-cell mixture was gently added to outgrowth medium and prewarmed to 37 °C. After 17–19 h, the cells were scraped, fixed, and permeabilized with Cytofix/Cytoperm (BD Biosciences, San Jose, California, USA) and stained with AF-647 conjugated human anti-HA (made in-house) and/or AF-488 conjugated human anti-NA (made in-house) antibodies. The proportions of HA- and NA-positive cells were enumerated by flow cytometry using a Fortessa flow cytometer (BD Biosciences).

### Mouse immunogenicity studies

Mouse studies were conducted at Biomodels LLC (Waltham, Massachusetts, USA). Female BALB/c mice, 6–8 weeks old, maintained at Biomodels LLC, were immunized (10 mice per group) with bilateral 50 µL intramuscular injections in the rear quadriceps on days 1 and 22. Three weeks after the second immunization, the mice were euthanized by CO_2_ asphyxiation, and blood was collected to evaluate the serum antibody response. To assess cell-mediated immunity, spleens were removed from each animal immediately after euthanasia. All experiments were approved by the Biomodels LLC Institutional Care and Use Committee (IACUC) under IACUC protocol #22-0624-1 and carried out in accordance with the National Institutes of Health Guide for the Care and Use of Laboratory Animals^48^.

### Ferret challenge studies

Outbred, castrated and de-scented male ferrets aged 6 months were purchased from Marshall Bio-Resources (North Rose, New York, USA). Ferret studies were completed at Labcorp Early Development Laboratories Inc. and were reviewed and approved by Labcorp Early Development Laboratories, Denver Site IACUC, protocol #0135-22. The animals were handled by trained personnel per the provisions of the Animal Welfare Act, principles of the NIH Guide for the Care and Use of Laboratory Animals, together with the internal procedures and policies of CSL Seqirus and Labcorp. An ABSL-2 vivarium with 12 h light/dark cycles and appropriate temperature and humidity was used to host the animals and the subsequent experimental procedures. Prior to the commencement of the experiments the animals were confirmed seronegative for influenza A and B viruses using standard hemagglutination inhibition (HAI) assays. Randomly grouped (*n* = 6 or 5 per group) influenza-free ferrets were immunized intramuscularly at the quadriceps using a volume of 0.5 mL at indicated timepoints (Fig. 6a). Ferrets were bled under light isoflurane anesthesia to obtain serum prior to each immunization and 3 weeks post-boost on day 42. On study day 48, the ferrets were intranasally challenged with 10^6^ median tissue culture infectious dose (TCID_50_) A/Delaware/32/2019 H3N2 influenza virus in a volume of 1 mL (0.5 mL per nostril). Following challenge, nasal and throat swabs were taken daily under light isoflurane anesthesia and animals were monitored for morbidity/mortality in accordance with the Animal Welfare Act and the Association for Assessment and Accreditation of Laboratory Animal Care International (AAALACi) guidelines. At the end of the study ferrets were euthanized after deep sedation with isoflurane via cardiac terminal bleed followed by intracardiac injection of Euthasol. Tissues were collected after confirmation of death and double thoracotomy.

### Viruses

The seasonal influenza viruses used in this study included A/Delaware/55/2019 (H1N1), A/Delaware/39/2019 (H3N2), B/Darwin/7/2019 (B/Victoria), and B/Singapore/INFTT-16-0610/2016 (B/Yamagata). To investigate NA-mediated cross neutralization, we used additional A(H3N2) strains, including Indiana/08/2018, A/North Carolina/04/2016, and A/Singapore/GP050/2015. All viruses were propagated in Madin-Darby canine kidney (MDCK) cells (proprietary 33016-PF, Seqirus) at 34 °C for 72 h. Working virus stocks were then characterized by sequencing HA, NA, and nucleoprotein (NP) and titrated. For the purposes of this study, these viruses were titrated by a fluorescent focus-based method and/or by obtaining a TCID_50_ using MDCK cells.

### Short-form microneutralization assays

The neutralization capacity of the sera was examined by a virus fluorescent focus-based SF MN assay developed in house against homologous vaccine strains^49^. Serial dilutions of receptor-destroying enzyme (RDE) treated heat-inactivated sera were preincubated with 1000-2000 foci-forming units (FFU) of virus per well and allowed to react for 2 h at 37 °C before monolayers of MDCK cells were inoculated. Following overnight incubation at 37 °C, the monolayers were fixed, and infected cells were stained for the nucleoprotein of influenza A (clones A1, A3 blend, MilliporeSigma, Burlington, MA, USA) or influenza B (clones B2, B4 blend, MilliporeSigma) and labeled with a goat anti-mouse IgG (H + L) secondary antibody conjugated to Alexa Fluor 488 (Invitrogen, Waltham, Massachusetts, USA). Fluorescent foci were imaged by an immunospot analyzer (Cellular Technology Limited, Beachwood, Ohio, USA) and quantified with Immunospot 7.0.12.1 software (Cellular Technology Ltd., Cleveland, Ohio, USA). The MN titer was determined using Excel software (Microsoft, Redmond, Washington, USA) by calculating the reciprocal of the dilution that caused a 60% reduction in viral foci versus the no serum controls.

### Long-form MN assay

To assess MN mediated by anti-NA antibodies, a hemagglutination quantification-based LF MN assay was used. Serial dilutions of previously RDE and heat-treated serum samples were mixed with an equal volume of influenza virus solution containing 100 TCID_50_ of A(H3N2) in U-Bottom 96-well plates in neutralization medium consisting of 33016 MDCK protein-free media (Gibco #041-94718 A, Thermo Fisher) and incubated for 1 h at 37 °C and 5% CO_2_. This serum-virus mixture was transferred onto confluent MDCK 33016-PF cell monolayers and incubated for an additional 1 h at 37 °C and 5% CO_2_. The inoculating medium containing sera and virus was removed, monolayers were washed twice with sterile phosphate-buffered saline (PBS) to remove unbound virus, and cells were incubated for 5 days (37 °C and 5% CO_2_) in the presence of serially diluted serum in neutralizing media supplemented with L-(tosylamido-2-phenyl) ethyl chloromethyl ketone (TPCK)–trypsin (#T1426, Sigma-Aldrich, St. Louis, MO, USA). Control wells containing virus and cells, virus back-titration, and cells without virus were included in each plate. After 5 days, the plates were examined by hemagglutination assay to determine the LF MN titers. Fifty microliters of supernatant were transferred to each well of specific rows into V-bottomed 96-well microtiter plates, an equal volume of 0.7% guinea pig red blood cells (GPRBC; Lampire Biological Laboratories, Pipersville, Pennsylvania, USA) was added, and the mixture was incubated at room temperature for 30 min. The presence of RBC agglutination (no neutralization) or absence (neutralization) was observed for the vaccine strain (A/Delaware/39/2019) as well as heterologous A(H3N2) strains. The reciprocal of the highest serum dilution that protected the cells from infection was taken as the neutralization titer.

### Enzyme-linked lectin assay

On day 42 postimmunization, sera were examined for NAI activity by enzyme-linked lectin assay (ELLA)^50^. Briefly, NA from the homologous or heterologous vaccine strains was mixed with serial dilutions of heat-inactivated sera in buffer containing 33.3 mM 2-(N-morpholino)ethanesulfonic acid (MES, pH 6.5; Alfa Aesar, Haverhill, Massachusetts, USA), 4 mM calcium chloride (KD Medical, Columbia, Maryland, USA), 0.5% Tween-20, and 1% bovine serum albumin (BSA) fraction V (Calbiochem, San Diego, California, USA) in plates coated with fetuin (25 µg/ml in PBS, Sigma-Aldrich). Following overnight incubation at 37 °C, the cleavage of sialic acid was detected by peanut agglutinin-horseradish peroxidase (HRP) conjugate (1 µg/ml in PBS, Sigma), the plates were treated with 3,3′,5,5′-tetramethylbenzidine (TMB; Rockland, Royersford, Pennsylvania, USA), and the reactions were stopped with 2 N sulfuric acid (Sigma-Aldrich). Absorbance was measured on a Synergy H1 plate reader (BioTek, Winooski, Vermont, USA). The NAI titer was determined by performing a nonlinear regression in GraphPad Prism (GraphPad Software, San Diego, California, USA) and calculating the reciprocal of the dilution that resulted in a 50% reduction in neuraminidase activity versus the no serum controls.

### T-cell antigen stimulation and intracellular cytokine staining

For T-cell analysis, spleens from immunized mice were pooled from 5 mice per group, and single-cell suspensions were prepared in Roswell Park Memorial Institute medium (RPMI, Gibco #22400, Thermo Fisher) containing 100 units of penicillin, 100 µg streptomycin, and 50 μM 2-mercaptoethanol. Duplicate cultures of 2 × 10^6^ splenocytes for each stimulation condition were prepared for each splenocyte pool, as well as unstimulated cultures that did not contain antigens but were otherwise identical to stimulated cultures. Influenza subtype-specific CD4 T cells were stimulated with homologous MDCK cell–derived monovalent influenza vaccine component, or monobulk, at a final concentration of 10 µg/mL. In the same wells, CD8 T cells were stimulated with peptides (1 µg/mL) representing influenza A H1 amino acids 533-541 for H1 (peptide IYSTVASSL) and conserved antigen 551-559 (peptide YYSTAASSL) for B/Victoria and B/Yamagata. All cultures contained anti-CD28 antibody (BD Biosciences #553294) at a final concentration of 1 µg/mL, and after stimulation for 2 h, BD GolgiPlug™ Protein Transport Inhibitor (containing Brefeldin A) (BD Biosciences #555029) was added. Stimulation was performed for a total of 6 h in a humidified incubator at 37°C (5% CO_2_). After stimulation, the cells were stained with a LIVE/DEAD^TM^ fixable aqua dead cell stain kit (Invitrogen #L34966), washed, and stained with APC-H7-labeled anti-CD4 (1:20, BD Biosciences #560181) and Alexa Fluor 700-labeled anti-CD8 (1:100, BD Biosciences #557959). Cells were washed, fixed with Perm/Wash buffer (BD Biosciences) and stained with a mixture of Brilliant Violet 605-labeled anti-IL-2 (1:80, BD Biosciences #563911), Alexa Fluor 488-labeled anti-TNFα (1:160, BD Biosciences #557719), PerCP/Cy5.5-labeled anti-IFN-γ (1:160, #45-7311-82, eBioscience, San Diego, California, USA), allophycocyanin-labeled anti-IL-5 (1:80, #504306, BioLegend, San Diego, California, USA), and phycoerythrin-labeled anti-IL-13 (1:80, eBioscience #12-7133-82). Flow cytometry was performed on a Fortessa (BD Biosciences) and analyzed by FlowJo software v10.8.1 (BD Biosciences). The net percent of Ag-specific CD4 or CD8 T cells was calculated as the difference between the percent cytokine-positive cells in the Ag-stimulated and unstimulated cultures. The 95% confidence limits for the percent Ag-specific cells were determined using standard statistical methods in Microsoft Excel. Reported values are the results of duplicate measurements on pooled spleens; therefore, these error bars represent the precision of the measurement, rather than variability within the group.

### Viral load determination using plaque assays

MDCK-33016-PF cells were seeded in 12 well cell culture plates in DMEM (Gibco #11960, Thermo Fisher) with 10% FBS and 1% penicillin-streptomycin-glutamine (Gibco #10378016, Thermo Fisher) a day before the assays. Homogenized and clarified tissues or clarified supernatants from swabs were subjected to tenfold serial dilutions in virus diluent (Opti-MEM; Thermo Fisher #31985070). Monolayers were washed twice with sterile PBS and inoculated with the serially diluted samples for 1 h while rocking the plates every 10 min to avoid drying out the cells. After virus adsorption, the inoculum was removed, and a semisolid overlay composed of Eagle’s minimum essential medium (EMEM; VWR #12001-584, Radnor, Pennsylvania, USA), 5% sodium bicarbonate (VWR #470302-440), diethylaminoethyl (DEAE) dextran (VWR #AAJ63781-14), and penicillin-streptomycin-glutamine with a final concentration of 0.7% of purified oxoid agar (Thermo Fisher #LB0028B) supplemented with TPCK-treated trypsin (1 µg/mL) was added to wells. The plates were incubated for 3 days at 37 °C with 5% CO_2_ and fixed using 4% PFA (Thermo Fisher #J199943) overnight. Viral plaques were stained using anti-influenza A virus NP antibody (MilliporeSigma #MAB8251) followed by an anti-mouse-HRP conjugated secondary antibody and developed using KPL TrueBlue reagent (#5510-0030, Seracare, Milford, Massachusetts, USA). Stained plaques were manually counted using a white light-transilluminator.

### Viral load determination using qRT-PCR

A(H3N2) viral load was determined by nucleic acid-based quantification^51^. Viral RNA (vRNA) extraction from homogenized-clarified tissue supernatants or clarified swab supernatants was performed using the QIAGEN vRNA extraction kit as per manufacturer’s instructions (#52906, Qiagen Inc, Valencia, California, USA). To determine the copy number, a purified and quantified molecular standard for A(H3N2) was obtained from ATCC (#VR-1882DQ) and was used to establish a standard curve using known copy numbers. vRNA extracted from samples was used to run the assay and copy numbers were established by extrapolation by the standard curve. The primers and probe sequences used for this study targeted the viral matrix (M) gene were Forward 5′-AGATGAGCCTTCTTACCGAGGTCG-3′, Reverse 5′-AGCAAAGACATCTTCAAGTCTCTG-3′, and Probe 5′-6 FAM- TCAGGCCCCCTCAAAGCCGA-TAMRA-3′ (Integrated DNA Technologies, Coralville, Iowa, USA). The qRT-PCR assay was performed using the Qiagen OneStep RT-PCR Kit (Qiagen #210212) and a Quantstudio 3.0 real-time thermal cycler (Thermo Fisher). All samples were run in triplicate with appropriate assay controls, and final values were normalized to appropriate volumes and/or weights.

### Statistics

For serological assays, log-transformed titers were compared between groups by two-way analysis of variance (ANOVA) with the Šidák multiple comparisons test using GraphPad Prism software (version 9.1.2, GraphPad Software). The GMT was plotted as a bar graph, and individual titers were plotted as points to show the distribution. To evaluate the magnitude of cross-neutralization in LF MN experiments, the neutralization titer for heterologous A(H3N2) strains in each serum sample was divided by the vaccine strain GMT to provide a percent homologous LF MN titer, which was plotted as the mean ± the standard error of the mean (SEM).

### Reporting summary

Further information on research design is available in the Nature Research Reporting Summary linked to this article.

### Supplementary information

Supplemental Material

Reporting Summary

## Data Availability

The authors declare that all relevant data supporting the findings of this study are available within the paper.
