# Supplementary material for: Self-amplifying mRNA seasonal influenza vaccines elicit mouse neutralizing antibody and cell-mediated immunity and protect ferrets
Source: NPJ Vaccines. 2023 Oct 4;8:150. doi: 10.1038/s41541-023-00747-2 (PMC10550923; doi:10.1038/s41541-023-00747-2)

Supplemental figures for - Self-amplifying mRNA seasonal influenza vaccines elicit mouse neutralizing antibody and cell-mediated immunity and protect ferrets

Michael Cheung[1#], Cheng Chang[1#], Raveen Rathnasinghe[1], Evan Rossignol[1], Yunfei Zhang[1], Annette Ferrari[1], Harsh Patel[1], Yanjun Huang[1], Michelle Sanchez Guillen[1], Tina Scalzo[1], Changkeun Lee[1], Gillis R. Otten[1], Ethan C. Settembre[1], Nedzad Music[1], Giuseppe Palladino[1], Yingxia Wen[*]

[1]CSL Seqirus, 225 Wyman Street, Waltham, MA 02451, USA
[#]Contributed equally

Supplementary Fig. 1. Bicistronic sa-mRNA vaccines induce both anti-HA and anti-NA IgG. a. HA-specific IgG antibodies induced by monovalent and quadrivalent sa-mRNA-HA-NA vaccines were measured by ELISA in vaccine strain monobulks. **b.** NA-specific IgG antibodies were measured by ELISA for recombinant NA proteins from each vaccine strain. mHA-NA and qHA-NA compared by 2-way ANOVA, * $p < 0.05$, ** $p < 0.01$, *** $p < 0.001$, **** $p < 0.0001$.

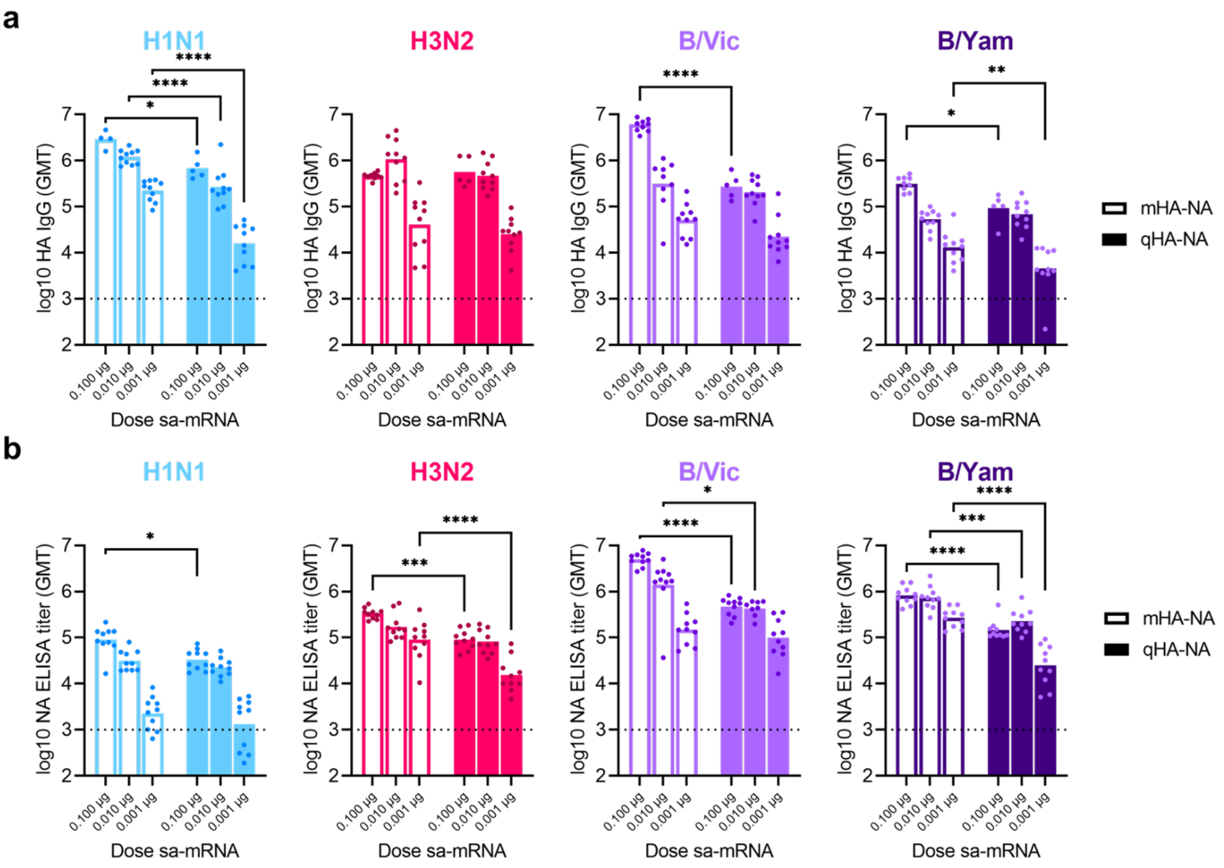

Supplementary Fig. 2. sa-mRNA vaccines establish vaccine-specific memory B cells in monovalent and quadrivalent formulations, as shown by the ELISPOT assay. Vaccine-specific memory B cells were detected in mice immunized with sa-mRNA vaccine (0.1 µg dose) by quantifying vaccine-specific antibody secreting cells in *ex vivo*-stimulated splenocytes 3 weeks following the second immunization.

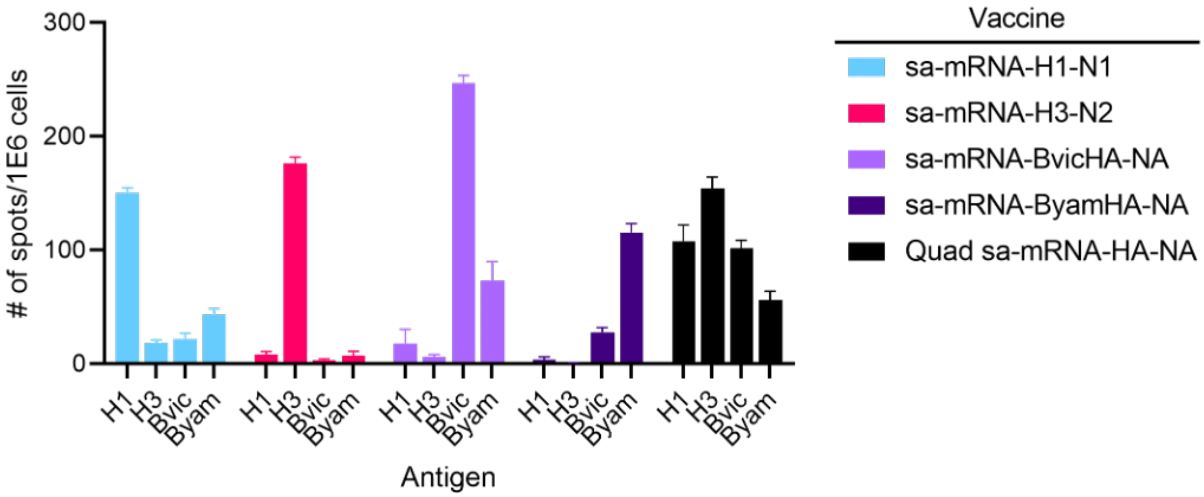

Supplementary Fig. 3. Quadrivalent formulations of either sa-mRNA or adjuvanted subunit vaccines result in lower titers than monovalent vaccines. The HA-specific neutralizing antibody response to 1 µg monovalent (open bars) and quadrivalent (filled bars) sa-mRNA-HA or adjuvanted subunit vaccines was measured by a short-form microneutralization (MN) assay. 2-way ANOVA, **p* < 0.05, ***p* < 0.01, ****p* < 0.001, *****p* < 0.0001.

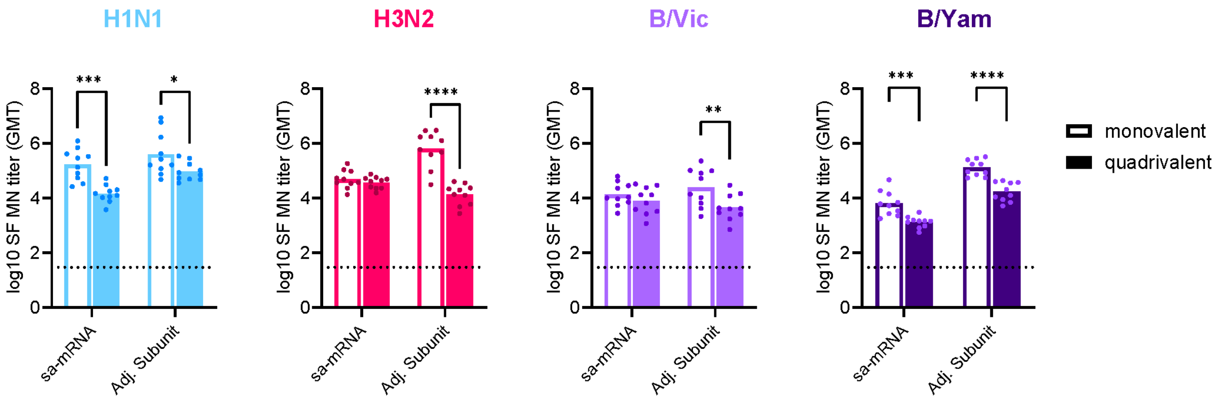

Supplementary Fig. 4. Phylogenic distance of HA and NA in recent A(H3N2) vaccine strains. a. A phylogenetic tree of H3 for the four A(H3N2) strains in Fig. 5 was generated using Geneious Prime software. **b.** A similar phylogenetic tree was created for N2.

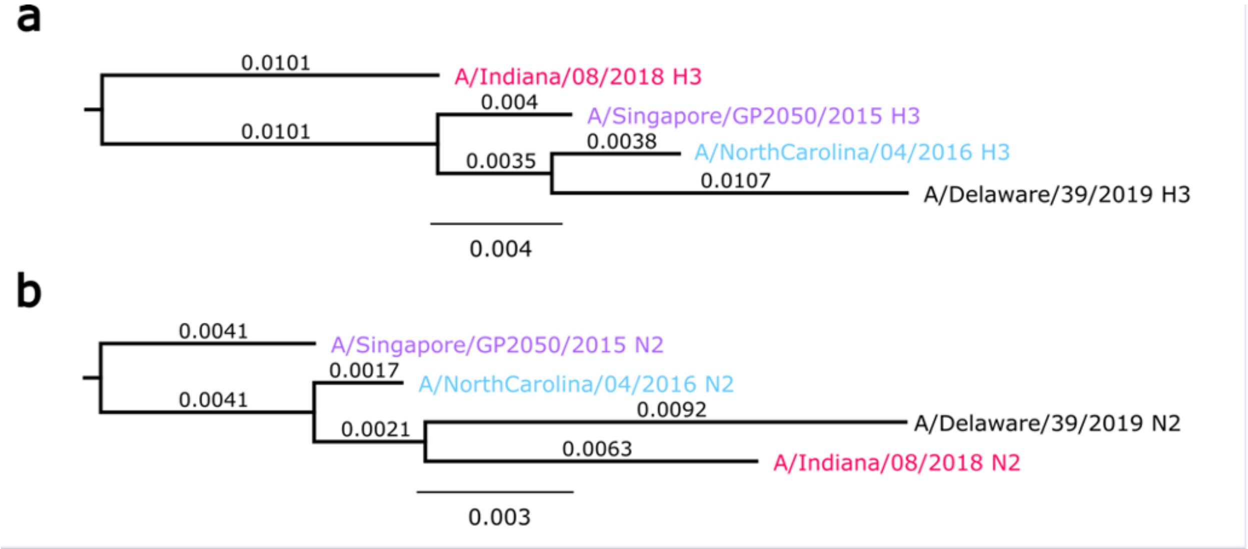

Supplementary Fig. 5. Bicistronic and monocistronic H3 and N2 sa-mRNA vaccines reduced viral load in throat and lung tissue of A(H3N2)-challenged ferrets. Viral load was quantified for daily throat swabs by plaque assay (**a**) and qRT-PCR (**b**). Viral load in homogenized lung lobes 3 days post challenge were determined by plaque assay (**c**) and by qRT-PCR (**d**). Each dot represents an animal and data are presented as geometric means. Data for daily throat swabs were log-transformed and analyzed using two-way ANOVA with multiple comparisons using Dunnett's adjustment. Lung tissue data was log-transformed and analyzed using a one-way ANOVA with multiple comparisons using Dunnett's adjustment. PBS, phosphate-buffered saline. $*p < 0.05$, $**p < 0.01$.

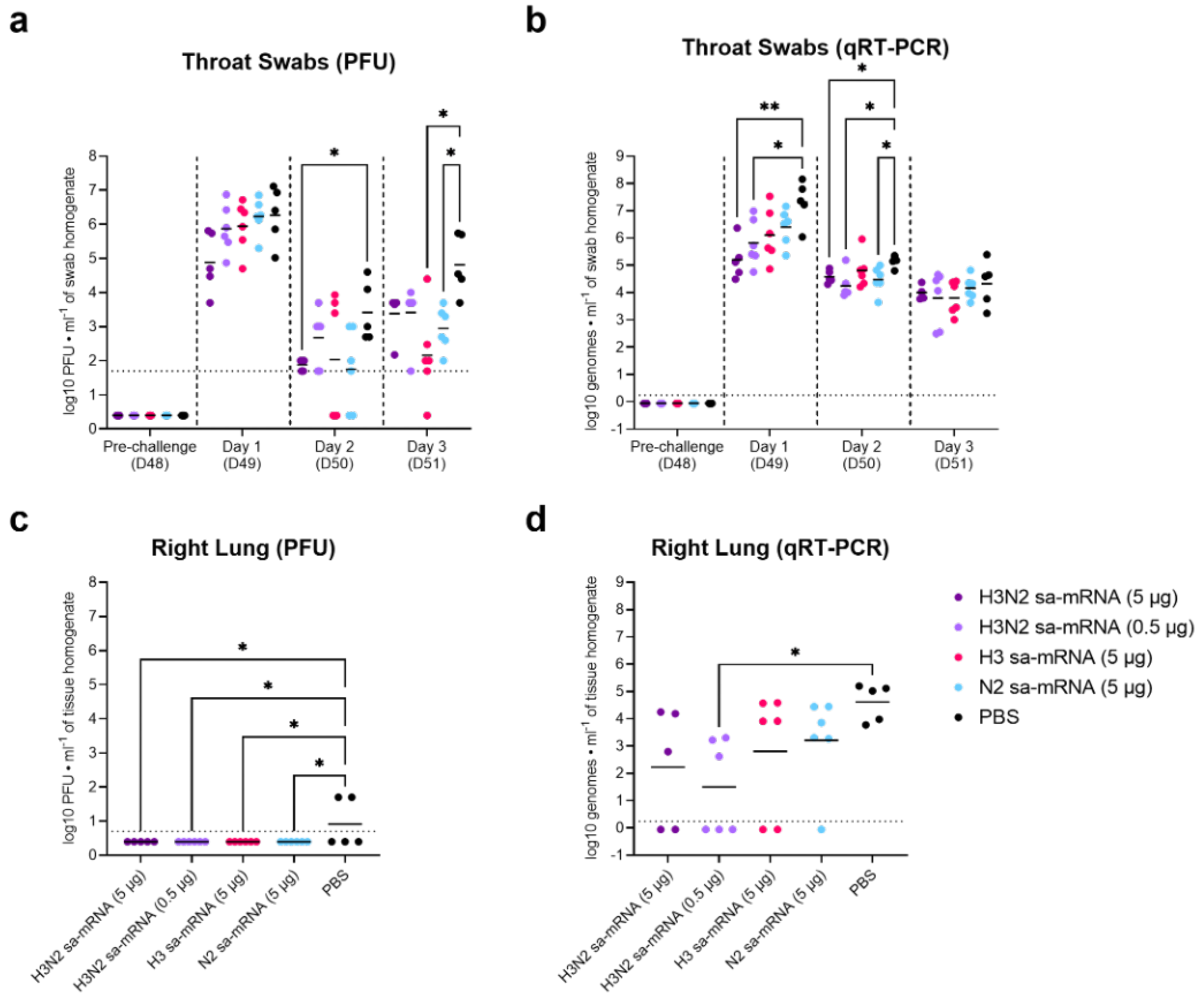

Supplementary Fig. 6. Bicistronic and monocistronic N2-containing sa-mRNA vaccines induced cross-neutralizing antibodies in ferrets. a. N2-specific neutralizing antibody response was measured by a long-form microneutralization (LF MN) assay. **b.** Cross-neutralizing response against heterologous A(H3N2) viruses are expressed as percent homologous (H3N2 A/Delaware/39/2019) LF MN titer (mean ± SEM). Titers against heterologous strains compared to A/Delaware/39/2019 by 2-way ANOVA, $^{**}p < 0.01$, $^{***}p < 0.001$, $^{****}p < 0.0001$.

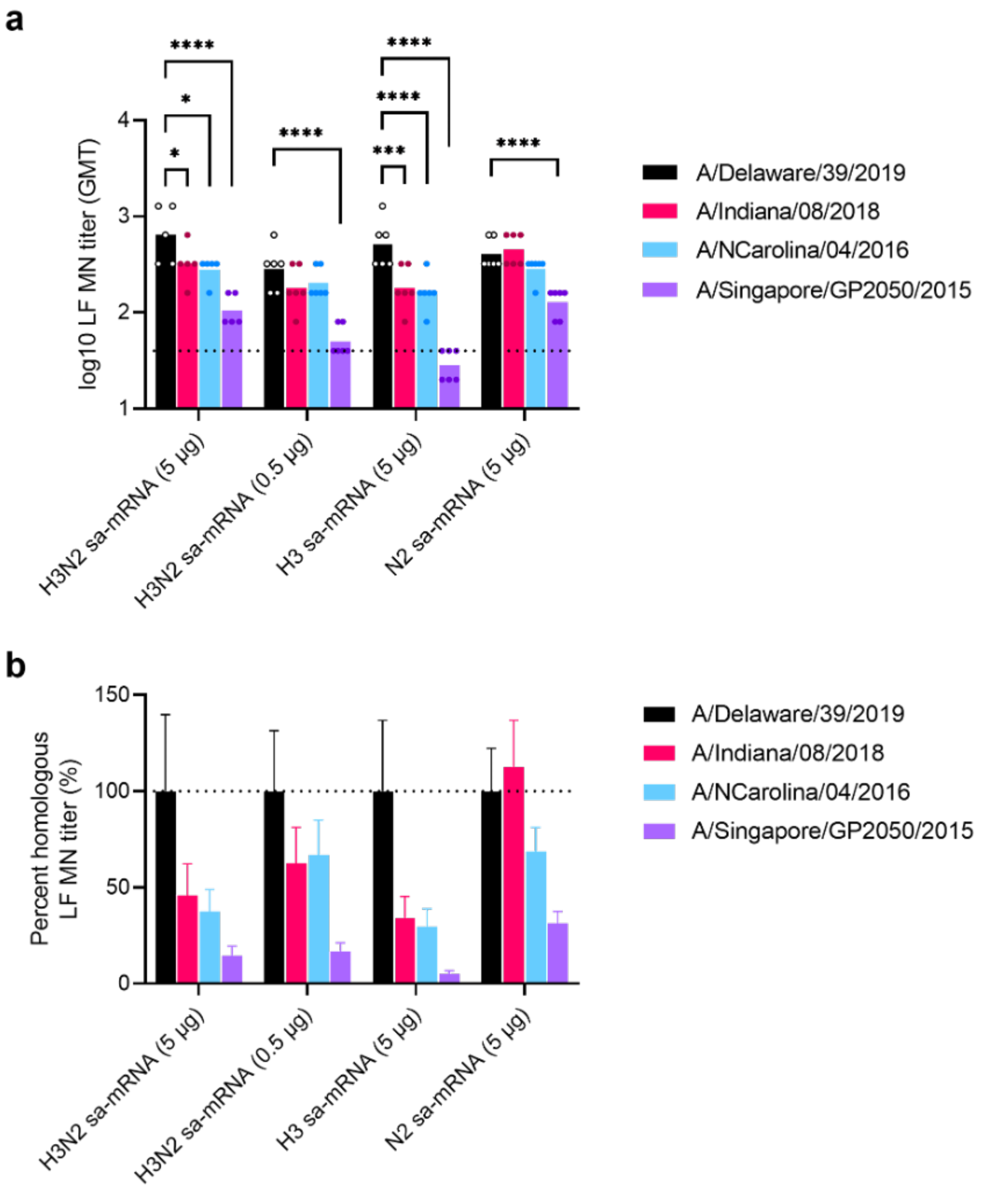

Supplement: Supplementary file 1 — Supplemental Material [file 41541_2023_747_MOESM1_ESM.pdf]
